# The Effect of Simultaneous Si and Ti/Mo Alloying on High-Temperature Strength of Fe_3_Al-Based Iron Aluminides

**DOI:** 10.3390/molecules25184268

**Published:** 2020-09-17

**Authors:** Věra Vodičková, Martin Švec, Pavel Hanus, Pavel Novák, Antonín Záděra, Vojtěch Keller, Petra Pazourková Prokopčáková

**Affiliations:** 1 Department of Material Science, Faculty of Mechanical Engineering, Technical University of Liberec, 461 17 Liberec, Czech Republic; vera.vodickova@tul.cz (V.V.); pavel.hanus@tul.cz (P.H.); vojtech.keller@tul.cz (V.K.); petra.prokop@tul.cz (P.P.P.); 2 Department of Technology, Faculty of Mechanical Engineering, Technical University of Liberec, 461 17 Liberec, Czech Republic; 3 Department of Metals and Corrosion Engineering, University of Chemistry and Technology, 166 28 Prague, Czech Republic; panovak@vscht.cz; 4 Department of Foundry Engineering, Brno University of Technology, 601 90 Brno, Czech Republic; zadera@fme.vutbr.cz

**Keywords:** Fe-Al-Si cast alloys, Ti and Mo alloying, long-term annealing, microstructure, high-temperature strength in compression

## Abstract

The effect of phase composition and morphology on high-temperature strength in the compression of Fe-Al-Si-based iron aluminides manufactured by casting was investigated. The structure and high-temperature strength in the compression of three alloys—Fe28Al5Si, Fe28Al5Si2Mo, and Fe28Al5Si2Ti—were studied. Long-term (at 800 °C for 100 h) annealing was performed for the achievement of structural stability. The phase composition and grain size of alloys were primarily described by means of scanning electron microscopy equipped with energy dispersive analysis and Electron Backscatter Diffraction (EBSD). The phase composition was verified by X-ray diffraction (XRD) analysis. The effect of Mo and Ti addition as well as the effect of long-term annealing on high-temperature yield stress in compression were investigated. Both additives—Mo and Ti—affected the yield stress values positively. Long-term annealing of Fe28Al5Si-X iron aluminide alloyed with Mo and Ti deteriorates yield stress values slightly due to grain coarsening.

## 1. Introduction

The iron aluminides with Fe_3_Al matrix are considered to be candidates for structural applications (e.g., turbine blades, glass furnace components, or grates of furnaces for heat treatment) mainly because of their excellent resistance to oxidation and sulfidation, as well as lower density. These alloys are also readily available for industrial use due to the low cost of feedstock in conventional casting.

The high-temperature mechanical properties of Fe_3_Al-based iron aluminides can be enhanced by strengthening by the solid solution hardening, e.g., by an addition of ternary elements such as Cr, V, Si, and Ti with a higher solubility in matrix. Furthermore, this can occur by strengthening due to the formation of incoherent or coherent precipitates, or by the increase of crystallographic order. The strengthening by coherent precipitates is known to be one of the most effective strengthening methods (in the Fe-Al-Ni system for example), but the strengthening effect at high temperatures can lead to a loss of ductility of alloys at low temperatures. In systems where the solid solubility of the third element is limited, strengthening by incoherent precipitates is possible. In some ternary systems (e.g., in Fe-Al-Zr or Fe-Al-Nb), the intermetallic phases can precipitate. Moreover, in the presence of C or B, element(s) with low solubility can form incoherent carbide and boride precipitates [1].

In Fe_3_Al-based iron aluminides with Si addition, the strengthening by solid solution hardening should work due to the high solubility of Si in Fe_3_Al matrix (27 at.% at 900 °C) [2]. The positive effect of the silicon addition on the corrosion resistance of iron aluminides, as well as the effect on their creep resistance, has already been described [3], but there is currently little information available on the effect of silicon on high-temperature strength.

Regarding silicon influence, it is possible to find many more results from an investigation into the effect of silicon on the mechanical properties of Al-rich alloys, because the research into the Fe-Al-Si system started due to the importance of the commercial Al-rich alloys, when the effect of silicon and iron impurities on the properties of these alloys was studied.

In the context of iron aluminide-based materials, the Fe-Al-Si ternary system was later investigated [4,5]. In detail, the system was studied focusing on the microstructure of binary and ternary phases in the temperature range between 600 and 900 °C [6], and reinvestigated recently [7] for two isothermal sections and six vertical sections. The phases forming in the Fe-Al-Si system were more specified, and the presence of a new phase was described.

Unlike silicon, the solubility of molybdenum in the iron aluminide matrix is lower, leading to the formation of intermetallic precipitates. If the morphology and distribution of this phase is sufficiently fine in matrix, the precipitates can participate in matrix strengthening [3]. On the other hand, detailed studies of the Fe-Al-Mo system [8,9] showed that the limiting Mo solubility in the iron-rich alloys increases dramatically with the temperature. It is necessary to take into account that the mechanism of strengthening could be modified (to solid solution strengthening + strengthening by incoherent precipitates) by another alloying element (e.g., Si or C).

The effect of the Mo addition on phase composition and distribution, as well as order–disorder phase-transition temperatures of FeAl-based alloy were investigated [10]. The enhancement of mechanical properties at room temperature after heat treatment in comparison to as-cast alloys was described. It was also shown that molybdenum in combination with a small amount of zirconium addition can influence the creep-rupture strength of binary Fe_3_Al significantly [11].

For both molybdenum and titanium, the strengthening of Fe-Al-based alloys by incoherent precipitates is possible. In the ternary Fe-Al-Ti system, the Laves phase works as a strengthening phase, while in the Fe-Al-Mo system the other intermetallic phases—besides the Laves phase—can act [1].

In ternary Fe-Al-Mo alloys with D0_3_ matrix, the strengthening by stable Mo_3_Al precipitates in combination with metastable τ_2_ phase can occur. The precipitate hardening depends on the molybdenum content, temperature, and prior heat treatment. The values of yield stress in compression were influenced by the precipitation of Mo_3_Al phase in the grains and at the grain boundaries during compressive testing at higher temperatures [12].

Titanium is generally mentioned as an additive for the enhancement of high-temperature mechanical properties. The Al-Fe-Ti system has already been studied in great detail as part of titanium aluminides research. Very detailed investigation was done in [13,14,15]. Fe-Al-Ti alloys are in the centre of interest due to the possibility of combining the strengthening mechanisms with each other—meaning the strengthening by incoherent precipitates with strengthening by order [1]. In particular, L21-ordered Fe-Al-Ti alloys have shown high yield stress and creep resistance compared with other Fe-Al-based alloys [16].

As regards further relevant systems, the ternary diagram Fe-Si-Ti is a complicated system containing many ternary compounds, similar to Al-Fe-Si. This system was investigated focusing on the structure and properties of the ternary phase [17,18]. The quaternary system Al-Fe-Si-Ti has also been reviewed, for example in [19], but the available data there are mainly in the Al-rich corner. Recently, a detailed study of the Fe-rich part of the quaternary system Al-Fe-Si-Ti was presented in [2].

In recent years, the research in the field of intermetallics has focused mainly on Fe-Al-Si alloys prepared by the processes of powder metallurgy, for example on alloys prepared by mechanical alloying or mechanical alloying and spark plasma sintering [20,21,22]. The benefits of powder technologies are indisputable, but costs tend to be higher than those of alloy produced by standard methods.

This work is focused on Fe-Al-Si(-X) alloys manufactured by standard casting process. The aim of this article is to show the effect of a low amount of additives on mechanical properties of cast Fe-Al-Si(-X) alloys. These alloys should have the potential for massive use due to economic technology, the price of raw materials used, as well as mechanical properties at elevated temperatures (up to 800 °C).

## 2. Materials and Methods

### 2.1. Alloys Production

The investigated alloys were prepared by vacuum induction melting and casting. The nominal chemical composition of the alloys is given in Table 1.

The batches for manufacturing the alloys with nominal chemical composition in atomic percentage of Fe-28Al-5Si, Fe-28Al-5Si-2Ti and Fe-28Al-5Si-2Mo were weighed solely from pure metals. The concentration of elements in used raw materials was Al 99.995, Si, Ti, and Mo 99.9 wt. %. The ARMCO^®^ Pure Iron with low carbon content declared by the manufacturer was used for casting. A ceramic crucible made from pressed zirconia material ZC93i was used for melting the alloys. A medium frequency vacuum induction furnace was used for the melting process. The zircon crucible was annealed before the melt.

Each melt was cast into a rectangular metal mold. The inner parts of the molds were sprayed with an aerosol of Y_2_O_3_. Prior to the casting of the alloy, the preheating of the mold was performed. This preheating was carried out in an electric resistance furnace (with a delay at 500 ± 2 °C and subsequent decrease to the final temperature of the mold (200 °C) that was appropriate for casting).

The melting process was carried out in a medium frequency vacuum induction furnace with the maximum melting weight of about 900 g. The main part of the batch consisted of pure metal. The metal was gradually alloyed with other elements in a vacuum according to the oxygen affine series of elements. For each melt there was a pressure of 4 Pa in the vacuum furnace chamber. When the batch had been melted completely, the furnace was filled with argon gas to 60,000 Pa, and consequently, the alloy was cast into a mold in a protective atmosphere of argon. The cast molds were left in a closed furnace to minimize the cooling speed of the ingots. All technological and metallurgical procedures having been observed, each ingot surface was without defects.

### 2.2. Experimental Methods

The samples were oxide-polished by the suspension OP-S for the study of microstructure. The structure was observed by scanning electron microscopes (SEM) Tescan Vega 3 SBH and Zeiss Ultra Plus. Tescan Vega 3 SBH was used for the overview study and for EBSD measurement (process parameters: HV = 20 kV, step size 1 µm, measured area 1 × 1 mm). High-resolution SEM Zeiss Ultra Plus was used for the detailed study of secondary phases. The individual images were always taken at the same work distance, magnification, detector and high voltage, to ensure a good image comparability (relevant for all images taken by Tescan SEM or taken by Zeiss SEM). Carbon content measured by Energy Dispersive X-Ray Spectroscopy (EDX) was only indicative of all measurements with respect to the used analytic method (the total carbon content of the studied alloys—determined by an appropriate analytical method by means of G4 ICARUS Bruker combustion analyzer—was in the range of 0.01–0.02 at.% for all alloys). The volume fraction of phases (f_V_) was calculated using NIS Elements software. Hardness measurements were performed by means of Struers hardness tester at 10g load (HV0.01).

The alloys were investigated in two states—in as-cast state (samples marked “as-cast”) and after stabilization annealing at 800 ± 5 °C for 100 h (samples marked as “HT 800/100”). Heat treatment was performed in the vacuum furnace. Long-term annealing was chosen based on the expected maximum application temperature (up to 800 °C). The aim of this annealing was to verify the presence and stability of the phases during long-term use of the material in this temperature range.

The samples (prisms 6 × 6 × 8 mm) for high-temperature compression tests were prepared by spark machining. The compression yield stress σ_0.2_ was measured using TESTOMETRIC FS100CT at a temperature of 20, 600, 700 and 800 °C with accuracy ± 1 °C) with the initial strain rate 1.5 × 10^−4^ s^−1^.

## 3. Results and Discussion

### 3.1. The Structure of Investigated Alloys

**Figure 1:**
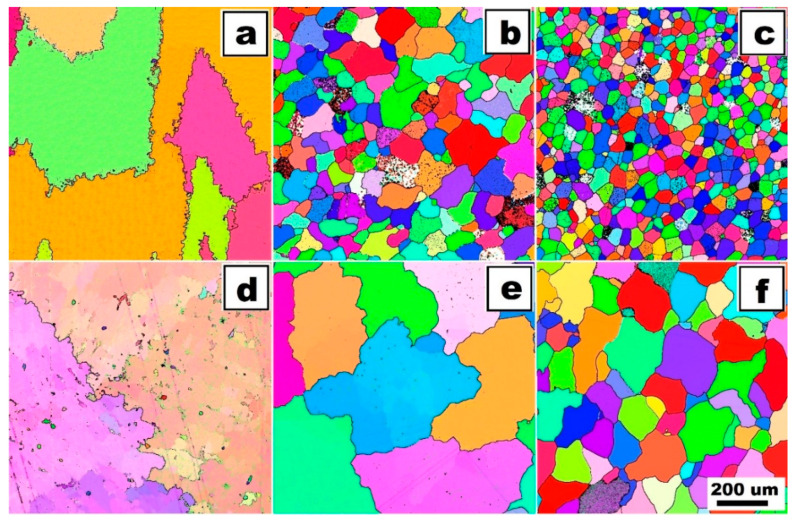
The dimensions of grains of investigated alloys by EBSD: (**a**) Fe28Al5Si as-cast, (**b**) Fe28Al5Si2Mo as-cast, (**c**) Fe28Al5Si2Ti as-cast, (**d**) Fe28Al5Si HT 800/100, (**e**) Fe28Al5Si2Mo HT 800/100, (**f**) Fe28Al5Si2Ti HT 800/100.

**Figure 2:**
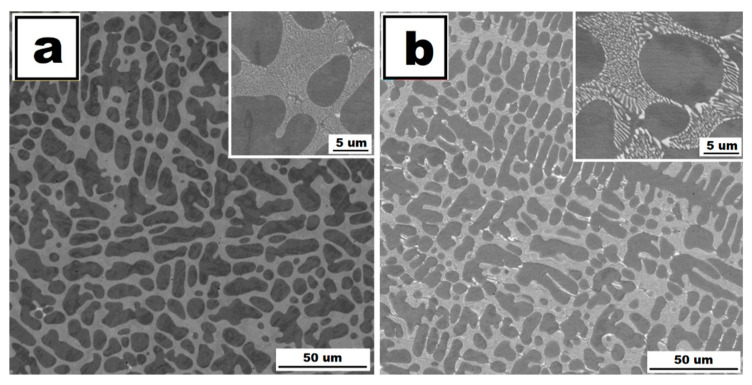
The structure of Fe28Al5Si alloy in as-cast state (**a**) and the structure of Fe28Al5Si alloy after annealing at 800 °C for 100 h (**b**)—(scanning electron microscopes (SEM), Back-scattered electrons contrast (BSE), 20 kV).

**Figure 3:**
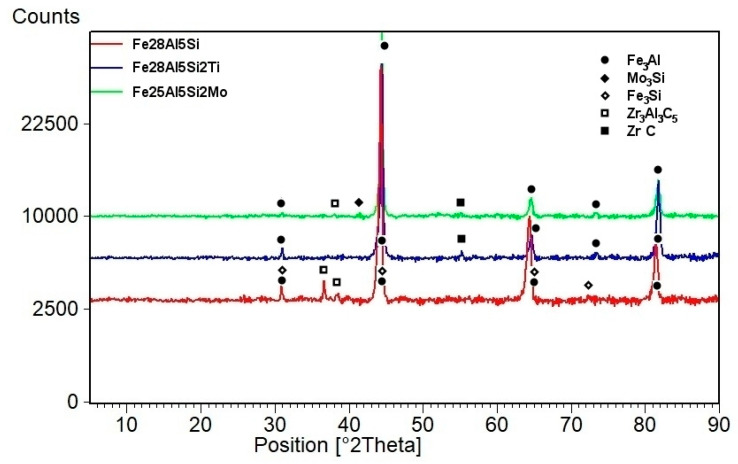
The evaluation of phases by XRD.

The structure of Fe28Al5Si alloy is coarse-grained in as-cast state (see EBSD map in Figure 1a). Dimensions of grains are in order of hundreds of micrometers. The matrix character is dendritic, inter-dendritic spaces are likely filled by eutectics (see Figure 2a). The phase composition of eutectics-like areas is very difficult to determine correctly with respect to the overlapping of peaks in XRD (Figure 3) as well as to the very small size of particles (in order of hundreds of nanometers). From the comparison of XRD to EDS results, it is possible to conclude that the eutectics-like areas are probably composed of Fe_3_Al matrix and a mixture of the complex carbides Zr_3_Al_3_C_5_ and Fe silicide (Fe_3_Si)—see XRD results in Figure 3. According to the ternary diagram and EDS measurements, silicon is also partly dissolved in the matrix (about 2 at.%).

The grains coarsened during heat treatment at 800 °C for 100 h (see Figure 1d). The dendritic arrangement of the structure remains after annealing (Fe28Al5Si HT 800/100)—see Figure 2b. However, the particles of secondary phases are coarser (locally with dimensions in order of units of micrometers). The eutectics are dissolved gradually during heat treatment. The chemical composition of matrix and particles did not change significantly after annealing.

**Figure 4:**
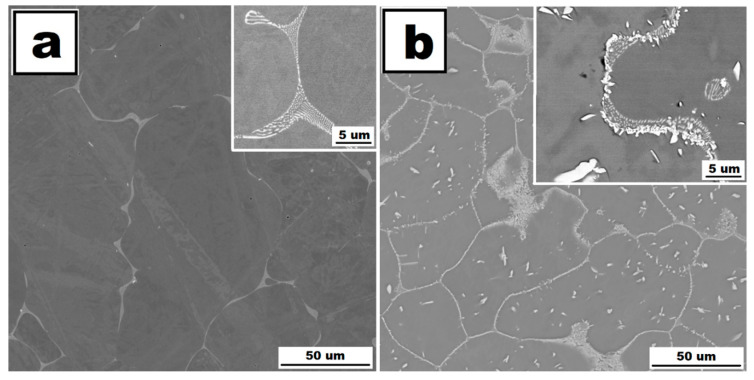
The structure of Fe28Al5Si2Mo alloy in as-cast state (**a**) and the structure of Fe28Al5Si2Mo alloy after annealing at 800 °C for 100 h (**b**)—(SEM, BSE, 20 kV). In detail (**b**): dark grey—Fe_3_Al matrix, light grey particles—eutectics, white particles—Mo-based silicides.

Molybdenum addition affects the grain size significantly. The grains are noticeably finer in Fe28Al5Si2Mo alloy in as-cast state in comparison to the alloy without Mo addition (see Figure 1a,b). The dimensions of irregularly shaped grains are in the range from approx. 50 to 200 µm. The areas of very fine residual eutectics (with particles in order of hundreds of nanometers) are distributed along the grain boundaries only. The composition of eutectic areas is similar to that of eutectics in the alloy Fe28Al5Si, where Zr carbides appear to be complex (Figure 3). Individual particles are also very rarely present inside the grains (see Figure 4a).

The heat treatment of 800 °C for 100 h has a significant effect on the grain size (see Figure 1e, compare with Figure 1b) and phase composition of Fe28Al5Si2Mo HT 800/100 alloy (see Figure 4b). Residual eutectics remains at the grain boundaries, however the particles of newly formed Mo-based silicide (Mo_3_Si according to XRD analysis, Figure 3) appear inside the grains and also along the grain boundaries, often together with original carbide eutectics (see detail in Figure 4b).

**Figure 5:**
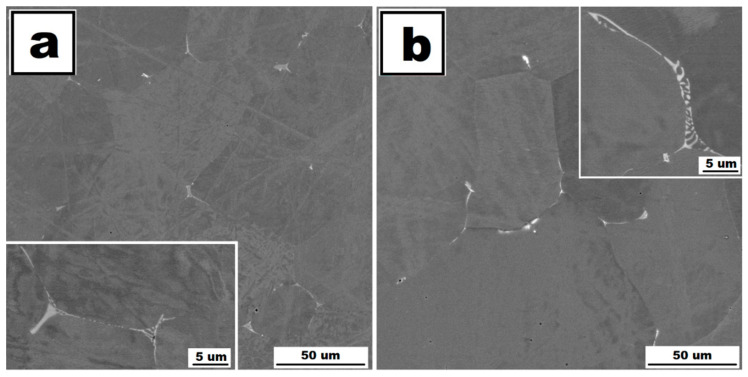
The structure of Fe28Al5Si2Ti alloy in as-cast state (**a**) and the structure of Fe28Al5Si2Ti alloy after annealing at 800 °C for 100 h (**b**)—(SEM, BSE, 20 kV).

The influence of the titanium addition on grain size is even more pronounced than that of molybdenum. Fe28Al5Si2Ti alloy in as-cast state has equiaxed grains with dimensions of tens of micrometres (see Figure 1c). The majority of the titanium addition is dissolved in the matrix, only a small part of Ti contributes to the formation of secondary particles present at the grain boundaries in the form of residual eutectics (see Figure 5a). This is composed of complex Zr carbides of Si or Ti (according to the comparison of EDS to XRD measurement, with respect to the small shift of diffraction peaks, Figure 3).

The annealing at 800 °C for 100 h does not lead to changes in the composition or morphology of the secondary phase (see Figure 5b). Eutectics is again distributed at the grain boundaries, and the size and shape of particles in eutectics stay the same. The grains are significantly coarser after annealing (see Figure 1f, compared with Figure 1c); however, the grain size is acceptable in comparison to the alloy without the quaternary additive.

### 3.2. The High-Temperature Yield Stress in Compression of Investigated Alloys

**Figure 6:**
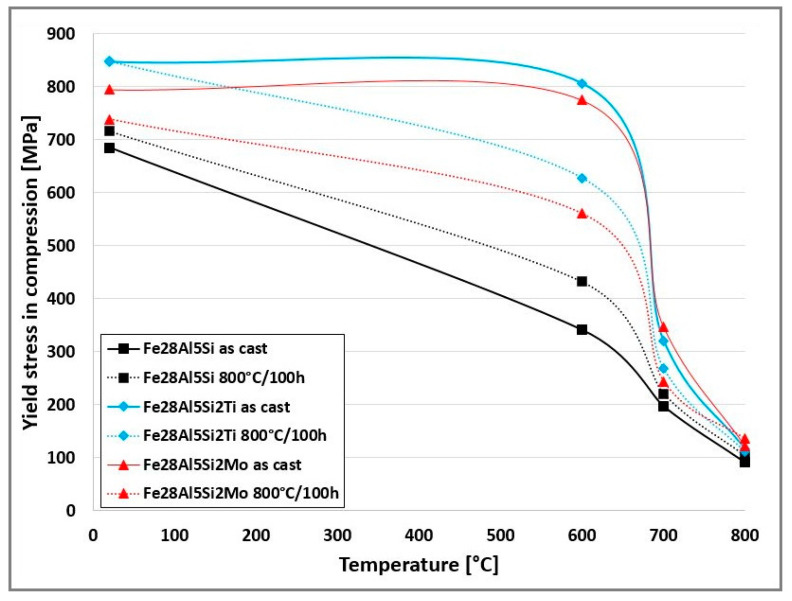
The temperature dependence of yield stress values for investigated alloys.

All the alloys based on Fe-Al-Si show high values of yield stress in compression at room temperatures (in range approx. 700–800 MPa depending on added quaternary alloying element). These yield stress values remain on a technically usable level even at high temperatures (700, 800 °C)—see Figure 6.

Both additives, molybdenum as well as titanium, increase the yield stress values significantly, especially at temperatures of 600 and 700 °C (see Figure 6).

The effect of annealing at 800 °C for 100 h on high-temperature yield stress in compression was also studied. The yield stress values increase slightly in the case of Fe28Al5Si alloy after heat treatment. The increase was in order of tens of MPa depending on temperature. However, the trend is quite the opposite in the case of the alloy Fe28Al5Si2Mo. The annealing leads to a significant decrease in yield stress values for all tested temperatures (decrease by up to 200 MPa at 600 °C). In the case of the alloy Fe28Al5Si2Ti, the value of yield stress is very similar to room temperature and to 800 °C. For the temperature of 600 and 700 °C the yield stress is higher for as-cast state (about 180 MPa at 600 °C and about 50 MPa at 700 °C)—see Figure 6.

### 3.3. Discussion

The dendritic character of the Fe28Al5Si alloy structure remains after heat treatment. EBSD measurements show that the grains are coarser after annealing at 800 °C for 100 h (compare Figure 1a,d). In spite of this fact, the yield stress values increase in the whole studied temperature range (especially at 600 °C). This can be caused by the gradual dissolution of eutectics into the matrix. This hypothesis was supported by the comparison of the volume fraction of the matrix and eutectics for both states of alloy as well as by the hardness measurement of the phases. The volume fraction of eutectics decreases after annealing and vice versa: the matrix hardness increases (see Table 2). Thus, it can be assumed that the cause of the matrix reinforcement is the eutectics dissolution.

A significant reduction in yield strength values in the case of the Fe28Al5Si2Mo alloy after annealing can be caused by the formation of a new type of secondary phase. This new phase—Mo_3_Si—is present along the grain boundaries and inside the grains. The finer silicides at the grain boundaries are located directly on primary eutectics areas. The silicides inside the grains are much larger (elongated shape with dimensions approx. 5 × 1 µm). Generally, the silicides neither at the grain boundaries nor inside the grains are able to contribute to strengthening due to their brittle behavior—it was shown that the mechanical properties of the stable Mo-based silicides depend on their stoichiometry [23]. The decrease in yield stress values at elevated temperatures can also be affected by significant coarsening of grains after annealing at 800 °C for 100 h.

The phase composition of Fe28Al5Si2Ti is not affected significantly by annealing. Additionally, yield stress values are comparable in both states, except yield stress values at 600 °C, which are significantly higher in the as-cast state.

**Figure 7:**
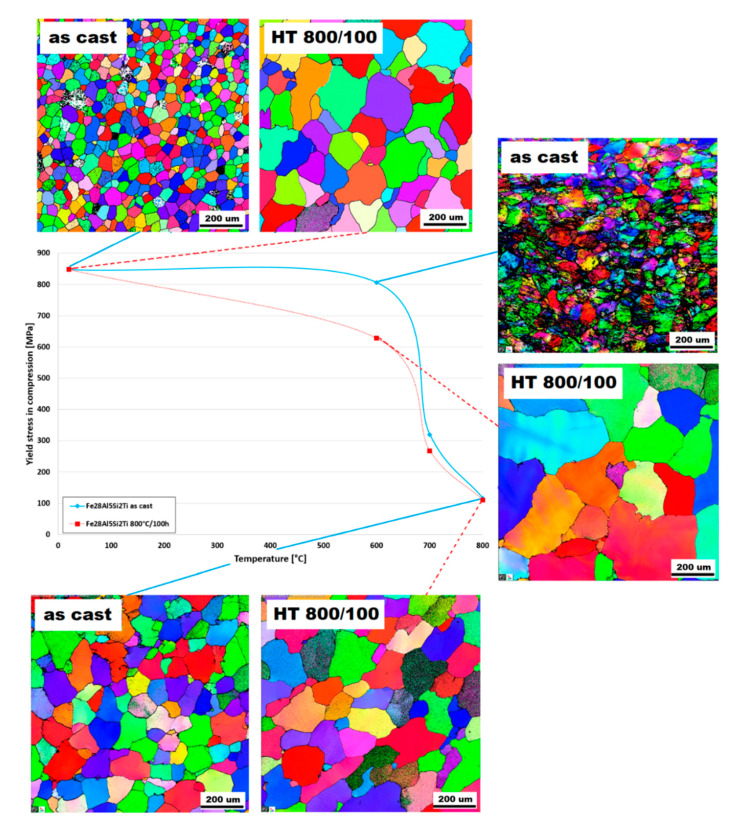
The comparison of grain size of Fe28Al5Si2Ti alloy after performed compression tests (the solid line corresponds to the as-cast state, the dashed line to the annealed state).

This fact corresponds very well with the changes in the grain size of the alloy Fe28Al5Si2Ti during high-temperature tests, as is shown in Figure 7. In the annealed state of the alloy, the grain size is significantly larger at 600 °C than that in the as-cast state, therefore the yield stress value of the annealed sample is almost 200 MPa lower at 600 °C. On the other hand, the grain sizes of both states are comparable at 800 °C and at room temperature, so the yield stress values are similar at these temperatures.

Both quaternary additives (Mo and Ti) were tested as potential strengthening elements for the enhancement of high-temperature mechanical properties in the past [12,24]. It was shown that they can contribute significantly to the strengthening of Fe_3_Al-based iron aluminides. The conditions of high-temperature compression tests [24] were the same as in this work. It is obvious from comparison [1,24] that the alloying with these additives to iron aluminides doped with silicon with Fe_3_Al matrix is even more effective. The yield stress values of quaternary Fe28Al5Si2Mo as well as Fe28Al5Si2Ti alloy are about two times higher than these of the ternary alloys Fe_3_Al-X (X = 2 at.% Ti or Mo) over the range of temperatures tested.

In this context, silicon in combination with Mo and especially with Ti appears to be a very efficient element for the enhancement of high-temperature strength in the compression of Fe_3_Al-based iron aluminides manufactured by the classic casting method.

The effect of different additives, titanium among others, on mechanical properties of Fe-Al-Si alloy prepared by mechanical alloying and spark plasma sintering was described in detail by Novak et al. [25]. It was showed, inter alia, that titanium addition improves the hardness values significantly, mainly due to the presence of hard phases. Based on the comparison of interplanar distances, it was also found that iron in the FeAl phase is partly substituted by titanium with a higher atomic radius. A similar mechanism could also contribute to strengthening and subsequently to improvement of high-temperature strength in the case of the alloyed alloys tested in this work.

## 4. Conclusions

Both additives, Ti and Mo, increase yield stress values of Fe28Al5Si alloy significantly.

Long-term annealing of 800/100 deteriorates the yield stress values of Fe28Al5SiMo and Fe28Al5SiTi alloys due to grain coarsening. Additionally, the formation of a Mo_3_Si as a brittle secondary phase during annealing can also contribute to the decrease in yield stress values in the case of Fe28Al5SiMo alloy. Conversely, the grain coarsening has no effect on the yield stress of Fe28Al5Si alloy, because the effect of strengthening by partial dissolution of particles of the secondary phase into the matrix is prevailing.

Alloying Si and Ti/Mo simultaneously shows a positive effect on the compressive yield stress values of Fe_3_Al-based iron aluminides, compared with Ti/Mo alloying individually. 

It can be assumed that a solid solution strengthening of Fe_3_Al matrix due to additive atoms, silicon and titanium mainly, contributes significantly to strengthening.

## Figures and Tables

**Table 1:** The nominal chemical composition of investigated alloys.

Alloy	The Nominal Chemical Composition [at.%]					
	Fe	Al	Si	Mo	Ti	Zr
Fe28Al5Si	Bal.	28.0	5.0	−	−	0.2
Fe28Al5Si2Mo	Bal.	28.0	5.0	2.0	−	0.2
Fe28Al5Si2Ti	Bal.	28.0	5.0	−	2.0	0.2

**Table 2:** Measured values of hardness and volume fraction of phases in both states of Fe28Al5Si alloy.

Alloy	Matrix Hardness [HV0.01]	Eutectics Hardness [HV0.01]	f_V_ of Eutectics [%]
Fe28Al5Si as-cast	455 ± 6	750 ± 4	44
Fe28Al5Si HT 800/100	549 ± 11	752 ± 9	34
